# An evaluation of a Shockroom located CT scanner: a randomized study of early assessment by CT scanning in trauma patients in the bi-located trauma center North-West Netherlands (REACT trial)

**DOI:** 10.1186/1471-227X-8-10

**Published:** 2008-08-22

**Authors:** Teun P Saltzherr, PH Ping Fung Kon Jin, Fred C Bakker, Kees J Ponsen, Jan SK Luitse, Mark Scholing, Georgios F Giannakopoulos, Ludo FM Beenen, C Pieter Henny, Ger M Koole, Hans B Reitsma, Marcel GW Dijkgraaf, Patrick MM Bossuyt, J Carel Goslings

**Affiliations:** 1 Trauma Unit Department of Surgery, Academic Medical Center, Amsterdam, The Netherlands; 2 Traumatology Department of Surgery, Vrije Universiteit medical Center, Amsterdam, The Netherlands; 3 Department of Radiology, Academic Medical Center, Amsterdam, The Netherlands; 4 Department of Anesthesiology, Academic Medical Center, Amsterdam, The Netherlands; 5 Department of Mathematics, VU University, Amsterdam, the Netherlands; 6 Department of Clinical Epidemiology and Biostatistics, Academic Medical Center, Amsterdam, The Netherlands

## Abstract

**Background:**

Trauma is a major source of morbidity and mortality, especially in people below the age of 50 years. For the evaluation of trauma patients CT scanning has gained wide acceptance in and provides detailed information on location and severity of injuries. However, CT scanning is frequently time consuming due to logistical (location of CT scanner elsewhere in the hospital) and technical issues. An innovative and unique infrastructural change has been made in the AMC in which the CT scanner is transported to the patient instead of the patient to the CT scanner. As a consequence, early shockroom CT scanning provides an all-inclusive multifocal diagnostic modality that can detect (potentially life-threatening) injuries in an earlier stage, so that therapy can be directed based on these findings.

**Methods/design:**

The REACT-trial is a prospective, randomized trial, comparing two Dutch level-1 trauma centers, respectively the VUmc and AMC, with the only difference being the location of the CT scanner (respectively in the Radiology Department and in the shockroom). All trauma patients that are transported to the AMC or VUmc shockroom according to the current prehospital triage system are included. Patients younger than 16 years of age and patients who die during transport are excluded. Randomization will be performed prehospitally.

Study parameters are the number of days outside the hospital during the first year following the trauma (primary outcome), general health at 6 and 12 months post trauma, mortality and morbidity, and various time intervals during initial evaluation. In addition a cost-effectiveness analysis of this shockroom concept will be performed.

Regarding primary outcome it is estimated that the common standard deviation of days spent outside of the hospital during the first year following trauma is a total of 12 days. To detect an overall difference of 2 days within the first year between the two strategies, 562 patients per group are needed. (alpha 0.95 and beta 0.80).

**Discussion:**

The REACT-trial will provide evidence on the effects of a strategy involving early shockroom CT scanning compared with a standard diagnostic imaging strategy in trauma patients on both patient outcome and operations research.

**Trial registration:**

ISRCTN55332315

## Background

Trauma is the most common cause of death in people younger than 50 years of age and accounts for more years of life lost than cancer, heart disease, and stroke combined. Injuries cause 5 million deaths every year worldwide (9% of global mortality).[1] In Europe alone injuries account for approximately 800,000 deaths (10% of all deaths) and 14% of all disability-adjusted life years (DALY).[2] Injuries are an important source of medical costs, economic losses, and immaterial losses. Trauma can therefore be regarded as a neglected epidemic.

For improving the trauma care specialized trauma centers are designated and specialized trauma care protocols, like the worldwide used ATLS^® ^guidelines, were developed.[3] Because there is a narrow window of opportunity between the moment that a patient deteriorates and actually dies, the ATLS guidelines prioritize care and focus on (potentially) life-threatening injuries rather than distracting but less important injuries. As a consequence a systematic approach of clinical examination and diagnostics is developed to recognize the most life-threatening injuries first. These should be treated immediately and preferably within 'the golden hour'.[3]

The imaging studies most frequently used in trauma patients include conventional X-rays, ultrasonography (FAST), and computed tomography scanning (CT). Although conventional X-rays and ultrasonography are widely used and easily accessible for many institutions, they have a limited sensitivity for injuries such as spine fractures,[4] pulmonary contusion, rib fractures, pneumothoraces or vascular injuries to the mediastinum, [5-8] and intra-abdominal, pelvic and retroperitoneal injuries.[9,10] Also, the amount of time necessary to obtain an overview of all the injuries is limited.

Recent improvements in CT technology with respect to image quality and speed have led to an increasingly important role of CT scanning in management of severely injured patients. However, the biggest problem with CT scanning is that this technique is frequently time-consuming due to logistical (location of CT scanner in other departments of the hospital) and technical issues. This implies that CT can only be used in hemodynamically stable patients where time to OR for surgical stabilization is a less critical factor. Furthermore, the fact that the same CT scanner is scheduled for elective patients as well as trauma patients means that an unplanned, prioritized trauma patient will disrupt the scheduled patient care and logistics and will lead to increased waiting times.

In order to improve patient care and workflow in acute trauma patients, the Academic Medical Center (AMC) in Amsterdam, the Netherlands, has initiated a project together with Siemens Inc. A new and revolutionary concept was developed in which the CT scanner is transported to the patient instead of the patient to the CT scanner. Main feature is a radiolucent trauma resuscitation table and a CT scanner that slides over the patient in the trauma resuscitating room. In addition there are also possibilities for conventional X-ray imaging and ultrasonography. With this concept the most important diagnostic modalities for trauma evaluation are available in the shockroom and CT scanning is possible at any moment during initial trauma evaluation. Furthermore, no further transport and patient transfers are required which can endanger the patient itself during the diagnostic phase (potentially leading to dislodgement of tubes, lines, cables, etc). Overall this concept will likely result in a faster and improved workflow and diagnostic imaging of trauma patients.

## Methods/design

### Study objectives

The primary objective is to prove the beneficial effects of early shockroom CT scanning on trauma patients by comparing the effects of a strategy involving early shockroom CT scanning with a standard diagnostic imaging strategy on patient outcome. In the latter strategy the CT scan is not located in the shockroom, but elsewhere in the hospital. The secondary objectives are to document the impact of introducing shockroom CT-scanning on logistics, capacity utilization, waiting times, economies of scale, substitution patterns, and investments.

### Study design

The REACT-trial is a prospective, patient-randomized study that will compare the clinical work-up of trauma patients in a setting where the CT scanner is located in the shockroom (AMC) with the standard situation where CT scanning takes place at the Radiology Department (VUmc).

### Setting/Participating centers

The Trauma Center North-West Netherlands is one of 10 designated Level-I trauma centers in the Netherlands. It is constituted by the 'Vrije Universiteit' medical center (VUmc) and the Academic Medical Center (AMC), which are both located approximately 8 kilometers apart from each other in Amsterdam. Each of these two hospitals, together with the surrounding affiliated hospitals, is responsible for the care of trauma victims in its region (2.7 million inhabitants in total) that are distributed over these hospitals. In both hospitals, patients are evaluated by a multidisciplinary team in the trauma resuscitation room ('shockroom'), which is fully equipped for initial management of trauma patients, including conventional X-rays and ultrasonography.

The initial evaluation of trauma patients after arrival is according to ATLS guidelines and the same in both hospitals. After the primary survey, standard X-rays (i.e. thorax, pelvis and cervical spine) and sonography will be done according to the ATLS guidelines.

In the VUmc the CT scanner (64-slice) is located in the Radiology Department on the second floor. This requires transportation of the patient with at least 4 patient transfers from trolley to the CT table and vice versa.

In the AMC a concept was developed in which the CT scanner is transported to the patient instead of the patient to the CT scanner. Main feature is a radiolucent trauma resuscitation table and a 4-slice CT scanner (SOMATOTOM Sensation 4, Siemens) placed on a rail which enables the scanner to slide over the patient. Because of a mirrored design of a second shockroom that is separated by radiation shielded sliding doors the CT scanner can be transferred over the rails into the second room after the imaging is finished. The first advantage of this design is that no interference is experienced from the CT scanner during trauma resuscitation. Secondly, the design allows simultaneous use of the mirrored trauma rooms, with the sliding CT scanner accessible to both rooms.

Both trauma resuscitating settings are equipped with a conventional X-ray installation and ultrasound. As a result of the AMC concept no further patient transport or transfers are necessary for obtaining a CT scan and all radiography can be performed in the trauma room. In addition, at any time during trauma resuscitation CT imaging can be performed.

### Endpoints

The primary outcome criterion used is the number of days spent outside the hospital in the first year following the trauma. This outcome is responsive to differences in mortality (no additional days outside hospital), to differences in hospital stay for the initial admission and to differences in readmission rate.

The secondary outcome parameters include general health outcome at 6 and 12 months after the shockroom admission (using the EuroQol and HUI-3 questionnaires), morbidity and mortality during the first year following the trauma and various time intervals and process of care parameters of the initial admission (time to intervention, time to active bleed management, ICU and total hospital stay, etc.). Furthermore the radiation dosage is calculated in both strategies based on the actual number and type of radiological examinations related to the initial trauma performed in each patient during the first year.

### Study group

All acute trauma patients are eligible for inclusion for the REACT trial when transported by the ambulance or helicopter to the AMC or VUmc shockroom according to the current pre-hospital triage system based on: Injury mechanism, Revised Trauma Score (RTS) and presence of traumatic brain injury. These factors determine the level of care that has to be present in the facility to which patients are transported.

The exclusion criteria for subsequent follow-up and analysis are patients younger than 16 years of age and patients who die during transport to the hospital.

The start of the study was scheduled for 1-11-2005.

### Randomization

Randomization will be performed at the "Meldkamer Ambulancezorg Amsterdam" (MKA), the organization in charge of the coordination and distribution of ambulances and patients. Randomization will be performed using a computer program on a 1:1 basis with varying block sizes of 8, 12, and 16. Ambulance personnel will receive instructions according to the outcome of the randomization. Each eligible patient involved in a specific accident will be randomized. After each randomization, there is a pre-specified time interval of 1 hour in which eligible patients will be automatically transported to the other trauma center in order to minimize peak pressure in the study centers and guarantee optimal utilization of the two trauma centers. These patients are included in the trial, but are not formally randomized. In extreme cases, prehospital ambulance personnel can decide to waive the outcome of randomization, if they deem that the status of the patient requires the immediate attention of the closest hospital and death is imminent.

Because the distance between the two hospitals is relatively short (8 km) no significant delay in treatment by patient transport is encountered regardless of the outcome of the randomization.

### Sample size

Based on the primary outcome criterion for both strategies, it is estimated that the common standard deviation is a total of 12 days. To detect an overall difference of 2 days in the number of days spent outside the hospital within the first year between the two strategies, 562 patients per group are needed for a two-sided significance level of 0.05 with a power of 0.80.

Based on historical data, we expect around 500 shockroom patients to be admitted on a yearly basis at each of the two participating centers. Therefore, the total number of eligible patients per year would be 1000 patients. We expect that a quarter of these patients will be excluded for various reasons (age < 16 yrs, lost to follow up, etc.) leading to a total of 750 inclusions per year. Consequently, a 1 1/2-year period should be sufficient to include the necessary total number of 1124 (2 × 562) patients.

#### Ethics and informed consent

The research protocol was primarily submitted to both the local Medical Ethics Committee (MEC) of the AMC and the VUmc to be reviewed. Both committees have been accredited to judge studies for the Central Committee on Research Involving Human Subjects and determined that the proposed study is not subject to the Medical Research Involving Human Subjects Act (WMO) and that therefore no further judgment is required for the study. Informed consents are not required from patients.

### Data analysis

The main analyses of primary and secondary outcomes will be conducted for all randomized patients according to the result of the randomization (intention-to-treat). Additional analyses include:

(1) Per-protocol analysis excluding patients that are transported to a different hospital rather than the result of the assignment procedure.

(2) Analysis of included patients treated either in the AMC or the VUmc independent of the randomization through the assignment procedure.

We will conduct both unadjusted and adjusted analyses. We will use gender, mechanism of trauma (sharp/blunt), initial Glasgow Coma Scale (GCS), RTS score and the presence of intubation to adjust for possible differences in severity of trauma between the AMC and VUmc patients.

For subgroup analysis the following analyses will be performed:

Hemodynamically unstable patients (non-responders (SBP < 90) vs. transient responders (SBP > 90 with continuous fluid requirement)); Sharp vs. blunt trauma patients; Patients prehospitally treated by Mobile Medical Teams; Neurotrauma patients; Presence or absence of a seatbelt sign; Torso trauma vs. isolated extremity trauma; Intubated vs. spontaneously breathing.

For final analysis standard statistical techniques will be used to compare the different outcomes between the two hospitals.

## Discussion

The REACT trial is a multicentered, prospective randomized trial that evaluates the effect of the newly introduced Amsterdam Trauma Workflow concept on trauma care. The main goal of the Amsterdam Trauma Workflow concept is to minimize the total diagnostic work-up time of the initial trauma evaluation by integrating all diagnostic modalities in the trauma resuscitating room. This concept makes it possible to perform CT imaging earlier during the trauma evaluation without the need to transport the patient to the radiology department. This adjustment will likely result in a faster and improved workflow of trauma patients, that leads to a more complete diagnostic workup in the early phases of trauma resuscitation. This could potentially change therapeutic management options and eventually lead to a better outcome in severely injured trauma patients. The direct availability of CT scanning during the entire trauma resuscitation phase could mean that this could become available for even hemodynamically unstable patients

A second advantage of this concept is the reduction in the number of patient manipulations, patient transfers and transports. Generally, these actions can have the aforementioned, adverse effects, which could expose the already critically ill patients to extra dangers. However, the introduction of the multifunctional radiolucent patient treatment table, that is suitable for resuscitation, conventional diagnostic imaging and CT scanning, minimize these actions and their additional risks.

While the REACT study design enables us to describe the diagnostic and therapeutic procedures following initial CT scanning on an individual patient level, the REACT trial also gives us the opportunity to evaluate its effect on an institutional level.

Because of the additional CT scanning capacity that was created by adding the sliding CT scanner that services the two mirrored trauma rooms, logistics for the radiology department will be influenced for both acute (trauma) patients and elective CT scanning. By potentially eliminating the need to reckon with unplanned acute CT imaging, the regular elective CT scans can be planned better and more efficient, possibly leading to a reduction of waiting times and waiting lists. Critically ill patient groups (i.e. trauma patients and patients with intracranial bleedings, acute aneurysms or abdomens, ICU patients, etc.) who need CT scanning can have their total diagnostic work-up completed in the shockroom before transport to their destination of treatment. In some cases the diagnostic work-up can even be completed simultaneously for two patients because of the mirrored shockroom design.

Furthermore, the REACT study design enables us to describe in detail the diagnostic and therapeutic procedures following initial CT scanning of trauma patients in the shockroom or at the Radiology Department. We may be able to demonstrate a trade-off in the volume and cost of health care use between early detection of injuries and timely therapeutic management on one hand and late detection by additional diagnostic testing and subsequent therapies on the other.

Another possible institutional effect might be that a shockroom CT scan may be used as an attractive alternative to other imaging procedures or to sequential diagnostic testing strategies in other patient groups (for instance stroke patients, patients with acute abdominal pain, etc), since it remains an all-inclusive multifocal diagnostic modality. As a consequence substitution of diagnostic modalities or changes in patient groups presenting for CT scanning may occur as a result of joint production.

Finally, the total costs of realizing this concept are of substantial amount and therefore this shockroom design has to be assessed during the study period in a cost-effectiveness analysis.

## Conclusion

The REACT trial is a prospective randomized multicenter trial that compares the effects of a new and revolutionary concept with a sliding CT scanner located in the trauma resuscitating room with a conventional setting, respectively a CT scanner located in the Radiology department. Results are expected early in 2009.

## Abbreviations

REACT: Randomized study of Early Assessment by CT scanning in Trauma patients, AMC: Academic Medical Center, VUmc: 'Vrije Universiteit' medical center. CT: Computed Tomography, FAST: Focused Assessment with Sonography in Trauma, SBP: systolic blood pressure, HUI-3: Health Utility Index 3, MKA: Meldkamer Ambulancezorg Amsterdam

## Competing interests

T.P. Saltzherr is a research fellow at the Trauma Unit Department of Surgery, employed by the AMC Medical Research B.V. and supported by a grant from Siemens Medical Solutions, Den Haag, the Netherlands.

## Authors' contributions

TPS drafted the manuscript. PHPFKJ and JCG co-authored the writing of the manuscript. All other authors participated in the design of the study and are local investigators at the participating centers.

## Pre-publication history

The pre-publication history for this paper can be accessed here:


